# Large scale enzyme based xenobiotic identification for exposomics

**DOI:** 10.1038/s41467-021-25698-x

**Published:** 2021-09-14

**Authors:** Ken H. Liu, Choon M. Lee, Grant Singer, Preeti Bais, Francisco Castellanos, Michael H. Woodworth, Thomas R. Ziegler, Colleen S. Kraft, Gary W. Miller, Shuzhao Li, Young-Mi Go, Edward T. Morgan, Dean P. Jones

**Affiliations:** 1 Clinical Biomarkers Laboratory, Department of Medicine, Emory University, Atlanta, Georgia USA; 2 Department of Pharmacology and Chemical Biology, Emory University School of Medicine, Atlanta, Georgia USA; 3 The Jackson Laboratory for Genomic Medicine, Atlanta, Connecticut USA; 4 Division of Infectious Disease, Department of Medicine, Emory University School of Medicine, Atlanta, Georgia USA; 5 Division of Endocrinology, Metabolism and Lipids, Department of Medicine, Emory University School of Medicine, Atlanta, Georgia USA; 6 Emory University School of Medicine, Department of Pathology and Laboratory Medicine, Atlanta, Georgia USA; 7 Department of Environmental Health Sciences, Columbia University Mailman School of Public Health, New York, New York USA

**Keywords:** Liquid chromatography, Metabolomics

## Abstract

Advances in genomics have revealed many of the genetic underpinnings of human disease, but exposomics methods are currently inadequate to obtain a similar level of understanding of environmental contributions to human disease. Exposomics methods are limited by low abundance of xenobiotic metabolites and lack of authentic standards, which precludes identification using solely mass spectrometry-based criteria. Here, we develop and validate a method for enzymatic generation of xenobiotic metabolites for use with high-resolution mass spectrometry (HRMS) for chemical identification. Generated xenobiotic metabolites were used to confirm identities of respective metabolites in mice and human samples based upon accurate mass, retention time and co-occurrence with related xenobiotic metabolites. The results establish a generally applicable enzyme-based identification (EBI) for mass spectrometry identification of xenobiotic metabolites and could complement existing criteria for chemical identification.

## Abstract (web summary)

Humans are exposed to many xenobiotic chemicals, but identification of low abundance xenobiotic exposures is limited by a lack of authentic standards for xenobiotic metabolites. Here the authors develop methods for enzymatic generation of diverse xenobiotic metabolites for use with high-resolution mass spectrometry for biology-based chemical identification.

## Introduction

Humans are exposed to tens of thousands of xenobiotic chemicals from the diet, drugs, environmental and occupational exposures, commercial products, and the microbiome^1^. Together with endogenously generated metabolites of the human metabolome, these are predicted to include over one million distinct chemical entities, representing a substantial analytical challenge for human exposome research^2,3^. To overcome the limitation in the number of chemicals measured with targeted methods, high-resolution mass spectrometry (HRMS) uses liquid chromatography with ultra-high resolution mass spectrometry and computational methods to measure chemicals as mass spectral signals without a priori knowledge of chemical identity^3^. This fills an important need to deliver -omics scale chemical data for environmental epidemiology to detect unrecognized health hazards^4^ but increases the need for additional approaches for chemical identification.

Xenobiotics are typically 3 to 5 orders of magnitude lower in abundance than endogenous metabolites and may be present in only a small fraction of human samples^5^. Confident identification of low-abundance xenobiotics is limited because mass spectrometry-based criteria require authentic standards and mass fragmentation spectra (MS^2^ or MS^*n*^)^6,7^. Standards are not available for most xenobiotic metabolites and only the top 1000–2000 highest intensity features have useful MS^2^ or MS^*n*^ spectra. Therefore, additional procedures are needed to complement accurate mass mass-to-charge ratios (*m*/*z*) for chemical identification.

Human enzymes convert xenobiotics to phase I and II metabolites. Usually, these related xenobiotic metabolites are expected to be present in samples with real exposures. With HRMS, computational approaches provide a strategy to test for co-detection of related xenobiotic metabolites by use of biological precursor–product relationships and metabolite–metabolite correlations. For example, multiple metabolites of naphthalene and another polycyclic aromatic hydrocarbon (PAH) were correlated and detected in military personnel exposed to burn pits^8^, providing credibility for PAH exposure despite signal intensities too low for MS^2^ analysis. Similarly, a metabolome-wide association study of the dichlorodiphenyltrichloroethane (DDT) metabolite chlorophenyl acetic acid identified two other correlated DDT metabolites^9^. While in silico tools for the prediction of xenobiotic metabolites^10^ and associated fragmentation spectra^11–13^ are available, the lack of readily available authentic standards for most xenobiotic biotransformation products limits our ability to identify xenobiotic metabolites in real samples.

In the present study, we develop a system to generate xenobiotic metabolites in a high-throughput manner to enhance mass spectrometry capabilities to identify xenobiotic metabolites in human samples. We use biological preparations with xenobiotic biotransformation enzyme activities (pooled human liver S9 fractions) to generate metabolites from a panel of xenobiotics, including some with known biotransformation products. We characterize metabolic products by accurate mass *m*/*z*, retention time, MS^*n*^, and stable isotope methods and match these to xenobiotic metabolites detected in mouse and human circulation. Our data show that related xenobiotic metabolites co-occur in human samples with documented exposures, and we apply this principle to identify undocumented environmental exposures. The results establish a method to provide authentic xenobiotic metabolites and associated stable isotopic forms for use with HRMS to improve confidence in the identification of low abundance xenobiotic metabolites in humans.

## Results

### Workflow for enzymatic generation of xenobiotic metabolites

**Fig. 1:**
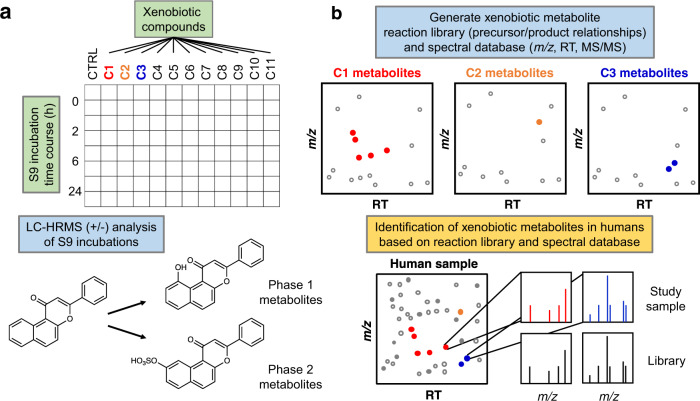
Enzymatic generation of xenobiotic metabolites for mass spectrometry-based chemical identification. **a** Human liver S9 enzyme prepared in a 96-well plate format capable of performing multiple xenobiotic reactions in a single plate. Human liver S9 enzymes perform Phase I and II biotransformation reactions to generate xenobiotic metabolites or other downstream adducts from reactive intermediates. **b** High-resolution mass spectrometry (HRMS) analysis of enzyme-generated xenobiotic metabolites provides authentic metabolites for matching accurate mass *m*/*z*, retention time (RT), and MS^2^ (if available) to detected metabolites in humans. Colored dots in each *m*/*z* RT dot plot represent spectral features that are identified from in vitro S9 reactions that could be matched to features detected in clinical or experimental studies. Co-occurrence of related xenobiotics in samples increases confidence in the identification of suspected exposure.

We aimed to develop a platform that enabled high-throughput enzymatic production of phase I and phase II xenobiotic metabolites. We used pooled human liver S9 fractions based upon previous findings that they are more representative of hepatocyte metabolism compared to microsomes and are more amenable for high-throughput screening applications compared to hepatocytes^14^. S9 fractions contain both microsomal and cytosolic subcellular fractions and include most phase I (cytochrome P450s, flavin monooxygenases, and aldehyde oxidases) and phase II (sulfotransferases, methyltransferases, glutathione S-transferases, N-acetyl transferases, and UDP-glucuronosyltransferases) biotransformation enzymes^15–21^. We prepared human liver S9 fractions with required cofactors in a 96-well plate format (Fig. 1a) which enabled the production of phase I and phase II metabolites (Fig. 1b) from multiple xenobiotics in a single plate. Reaction extracts were analyzed with HRMS to build retention time and spectral libraries for use with HRMS analyses of human samples (Fig. 1c).

### Production of phase I and II metabolites by human S9 enzymes

**Fig. 2:**
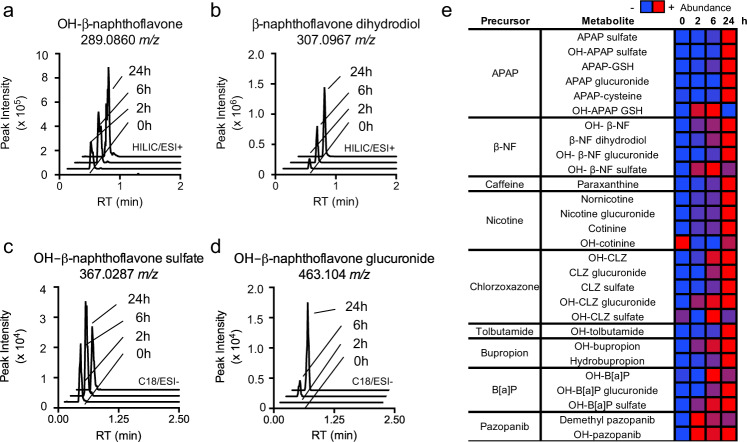
Human liver S9 enzymes generate Phase I and II xenobiotic metabolites in a time-dependent manner. Using standard conditions for S9 reactions, expected Phase I (**a**, **b**) and II (**c**, **d**) metabolites are formed in a time-dependent manner. Each plot depicts the extracted ion chromatograms of predicted metabolites of beta-naphthoflavone at 0, 2, 6, and 24 h. Metabolites were identified if they produced a time-dependent increase in MS^1^ signal corresponding to the accurate mass of a predicted or expected metabolite. **e** Formation of expected metabolites in a time-dependent manner for well-characterized xenobiotics using S9 enzymes.

To test whether standard conditions for S9 incubations could be used to produce biotransformation products of diverse xenobiotics, we tested 139 xenobiotics from environmental, pharmaceutical, and dietary/personal care products. Phase I and II metabolites for many (but not all) of these xenobiotics have been previously documented. Our data show that expected phase I and phase II metabolites were not detected at time 0 and increased with incubation time. For example, MS^1^ peak areas for hydroxy beta-naphthoflavone, beta-naphthoflavone dihydrodiol, hydroxy beta-naphthoflavone glucuronide, and hydroxy beta-naphthoflavone sulfate (Fig. 2a–d) increased with time and were not detected in the 0 h sample. Overall, more than 90% of expected metabolites from selected metabolites with well-characterized metabolism were detected in incubation extracts after 24 h (Fig. 2e). It is important to note that not all metabolites were detected at sufficient levels to collect mass fragmentation spectra. Extracted ion chromatograms and summarized data (MS^1^ *m/z*, adduct form, retention time, analytic method, MS^2^ if available) for other xenobiotic precursors are available online (http://metabolomics.cloud/#/project/CIDC001) and in supplementary data (Supplementary Fig. 1, Supplementary data 1). Raw files are available on Metabolomics Workbench (DOI: 10.21228/M8N97J). Thus, the present results show that the use of standardized human liver S9 fraction incubations is suitable as a platform for the production of expected phase I and phase II metabolites of diverse xenobiotics.

### Compound ID aided by isotope labels and prior knowledge

**Fig. 3:**
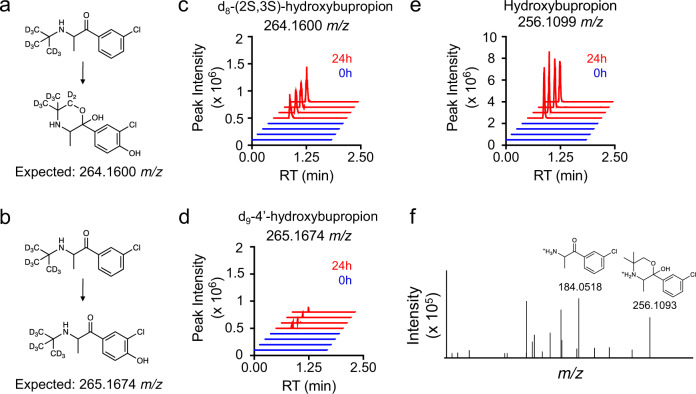
Stable-isotope assisted metabolite identification. Use of d_9_-bupropion with S9 enzyme system produces either **a** d_8_-(2S,3S)-hydroxybupropion or **b** d_9_-4′-hydroxybupropion. **c** Extracted ion chromatogram of 264.1600 *m*/*z* (d_8_-(2S,3S)-hydroxybupropion) at 0 and 24 h with the addition of d_9_-bupropion. **d** Extraction ion chromatogram of 265.1674 *m*/*z* (d_9_-4′ hydroxybupropion) at 0 and 24 h with addition of d_9_ bupropion. **e** Extracted ion chromatogram of 256.1099 *m*/*z* (hydroxybupropion) at 0 and 24 h with addition of bupropion. **f** Mass fragmentation spectrum (MS^2^) of 256.1099 *m*/*z* (black) is consistent with (2S,3S)-hydroxybupropion.

**Fig. 4:**
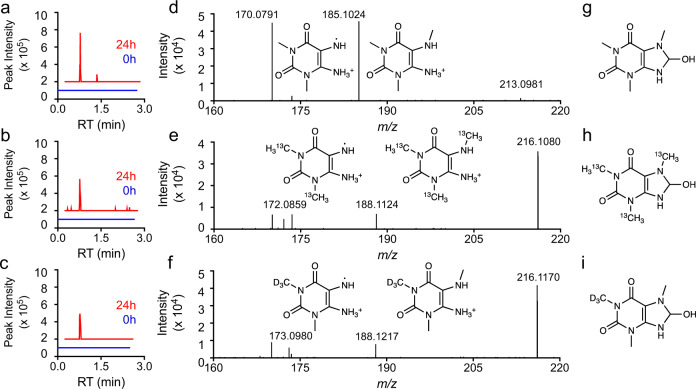
Stable isotope-assisted unexpected metabolite identification. **a** Time-dependent formation of 213.0981 *m*/*z* from caffeine. **b** Time-dependent formation of 216.1080 *m*/*z* from ^13^C_3_-caffeine. **c** Time-dependent formation of 216.1170 *m*/*z* from d_3_-caffeine. Annotated MS^2^ spectra of **d** 213.0981 *m*/*z*, **e** 216.1080 *m*/*z*, **f** 216.1170 *m*/*z*. Fragment interpretations are provided alongside labeled peaks. Proposed structures of unreported caffeine metabolite: **g**–**i** 213.0981 *m*/*z*, 216.1080 *m*/*z*, and 216.1170 *m*/*z*.

To further characterize S9-enzyme generated metabolites, we used isotopically labeled xenobiotic precursors to aid structure elucidation. Bupropion is mainly metabolized by CYP2B6, which performs a stereospecific hydroxylation to form (2S,3S)-hydroxybupropion^22^. However, 4′-hydroxybupropion, which shares the same exact mass, is also an expected minor product. Using d_9_-bupropion with S9 enzymes, we can differentiate between the formation of (2S,3S)-hydroxybupropion (Fig. 3a) and 4′-hydroxybupropion (Fig. 3b). (2S,3S)-hydroxybupropion is a heterocycle which loses one deuterium; whereas, 4′-hydroxybupropion is formed without loss of the deuterium label. Our data show the major product is d_8_-(2S,3S)-hydroxybupropion (264.1600 *m*/*z*) (Fig. 3c), with peak intensities, 10× higher than d_9_-4′-hydroxybupropion (265.1666 *m*/*z*) after 24 h (Fig. 3d). Using unlabeled bupropion, 256.1099 *m*/*z* alone does not discriminate between (2S,3S)-hydroxybupropion and 4′-hydroxybupropion (Fig. 3e). MS^2^ spectra collected from 256.1099 *m*/*z* are consistent with (2S,3S)-hydroxybupropion since we observe a fragment 184.0518 *m*/*z* (C_9_H_11_ClNO+) as opposed to an expected 200.0473 *m*/*z* (C_9_H_11_ClNO_2_+) if the oxidation occurred on the aromatic ring (Fig. 3f).

### Identification of unreported metabolites with stable isotopes

Full scan data collection for HRMS enables untargeted analysis to identify potential unreported metabolites. Here, we tested whether other spectral features that increased with time of S9 enzyme incubation could be unreported xenobiotic metabolites. To distinguish nonspecific reaction products from reaction products of test compound(s), we implemented an additional filter to account for mass differences due to labeling. For example, in the unlabeled caffeine reaction, 485 features were positively associated (*R* > 0.9) with time. Of these 485 features, we were able to identify two metabolites that were similarly increased with the addition of isotopically labeled (^13^C_3_ or D_3_) caffeine. Paraxanthine is the expected major product of caffeine metabolism and we observed increases in both unlabeled (181.0720 *m*/*z* 31 s RT) and labeled (^13^C_2_—183.0786 *m*/*z* 31 s RT, D_3_—184.0909 *m*/*z* 31 s RT) forms. Then, we identified an unexpected metabolite of caffeine with 213.0981 *m*/*z* (Fig. 4a, unlabeled, 31 s RT) with the formation of the isotopically labeled forms (^13^C_3_—216.1080 *m*/*z* (Fig. 4b), D_3_—216.1170 *m*/*z* (Fig. 4c)) at the same retention time. The metabolite 213.0981 *m*/*z* corresponds to an elemental composition C_8_H_12_N_4_O_3_, which corresponds to the addition of two hydrogens and one oxygen atom. MS^2^ analysis of 213.0981 *m*/*z* (Fig. 4d), 216.1080 *m*/*z* (Fig. 4e), and 216.1170 *m*/*z* (Fig. 4f) were consistent with oxidation (+O) and reduction (+2H) or hydration (+H_2_O) of the caffeine imidazole. A proposed structure for this unreported caffeine metabolite is provided (Fig. 4g–i). This metabolite was detected in mice treated with caffeine (Supplementary Fig. 2a) and also correlated with caffeine in humans (Supplementary Fig. 2b).

### Identification of documented xenobiotic exposures

**Fig. 5:**
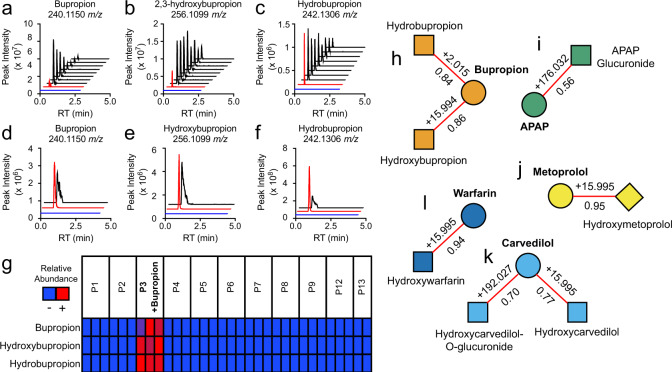
Identification of documented xenobiotic exposures. Extracted ion chromatograms of blood pharmacokinetic data from mice (black) that received bupropion show **a**–**c** bupropion, hydroxybupropion, and hydrobupropion are detected at the same accurate mass *m*/*z* and retention time as S9-enzyme generated metabolites (red). These metabolites were not detected in a control sample (blue). Extracted ion chromatograms of plasma collected from an individual (black) taking bupropion shows **d**–**f** bupropion, hydroxybupropion, and hydrobupropion are detected at the same accurate mass *m/z* and retention time as enzyme-generated metabolites (red). **g** Co-occurrence of bupropion with its expected metabolites only in the samples with documented bupropion use. Relative abundance values were colored across each row using the maximum observed peak intensities (red) and minimum observed peak intensities (blue) for a particular metabolite. **h**–**k** Pathway-level biotransformation networking of parent xenobiotics shows expected metabolites with characteristic mass shifts (above red edge) are correlated (Partial least squares regression *R* value shown below red edge) with parent xenobiotic.

Analysis of S9 reaction extracts provides authentic xenobiotic metabolites for generation of MS^1^, MS^2^, and retention times, which can support the identification of xenobiotic metabolites in human and mouse samples. Two of the criteria required for metabolite identification in human and mouse samples can be satisfied by the use of *m*/*z* (±3 ppm) and retention time windows (±5 s) for matching compounds detected in study samples. If MS^2^ cannot be collected for the study samples due to low abundance, the third criterion for identification can be satisfied if expected ions at their characteristic retention times are detected only in animal or human samples with documented exposure. For example, in mice treated with bupropion, bupropion (Fig. 5a), hydroxybupropion (Fig. 5b), and hydrobupropion (Fig. 5c) are detected at the same accurate mass *m*/*z* and retention time as S9-generated metabolites.

To test this concept in human samples, we used plasma samples collected from patients with well-documented pharmaceutical use (electronic medical records) receiving care at Emory University Hospital. HRMS analysis shows that in samples collected from the one individual who was taking bupropion, bupropion (Fig. 5d), hydroxybupropion (Fig. 5e), and hydrobupropion (Fig. 5f) were detected at the same accurate mass *m*/*z* and retention time as S9-generated metabolites. Furthermore, we observed that hydroxybupropion, and hydrobupropion were only detected in the samples where bupropion was also detected (Fig. 5g). Acetaminophen, carvedilol, warfarin, and metoprolol were other medications that were used by individuals in this cohort. To evaluate whether the expected metabolites of these medications were correlated with the initial parent compound, we used *x*MWAS software, a tool for multi-parameter metabolome-wide association analyses. Then, we applied a mass difference filter (*m*/*z*_feature_ − *m*/*z*_parent_) on correlated metabolites to generate reaction-based networks. Partial least squares regression analysis (*r* > 0.4) revealed five distinct clusters, each centered around a single parent compound. Each parent compound (bupropion Fig. 5a, acetaminophen Fig. 5i, metoprolol Fig. 5j, carvedilol Fig. 5k, warfarin Fig. 5l) was correlated with at least one expected biotransformation product and their associated source fragments, adducts, and isotopes (Supplementary Fig. 3). These metabolites were all detected at the same accurate mass *m*/*z* and retention times as those produced from S9 reactions. Thus, the use of enzyme-generated xenobiotic metabolites as authentic metabolites together with the use of co-detection of related metabolites provides a strategy for pathway-level identification of xenobiotics and xenobiotic metabolites.

### Identification of undocumented xenobiotic exposures

**Fig. 6:**
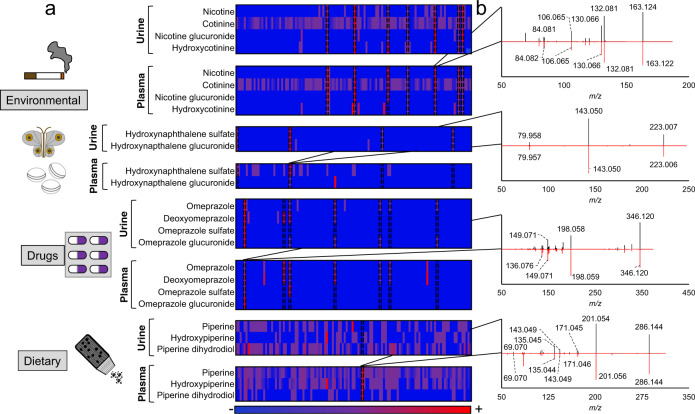
Identification of undocumented xenobiotic exposures. **a** Heatmap of relative peak intensities of related xenobiotics in 120 humans (each column from left to right represents an individual sample) with paired urine and plasma samples. Metabolites were detected at the same accurate mass *m*/*z* and retention time as S9-enzyme generated metabolites. Relative abundance values were colored across each row using the maximum observed peak intensities (red) and minimum observed peak intensities (blue) for a particular metabolite. **b** MS^2^ spectra of selected metabolites (nicotine, hydroxynapthalene sulfate, omeprazole, piperine) in experimental sample (black) compared with S9-reactions (red).

To test whether the use of accurate mass *m*/*z*, RT, and co-detection of related xenobiotics could be used for identification of xenobiotics in human samples with undocumented environmental, drug, or dietary exposures, we examined a healthy cohort (*n* = 120) with no documented xenobiotic exposures where paired urine and plasma samples were available. Nicotine, a common environmental xenobiotic that humans can be exposed to through tobacco products and secondhand smoke, was detected alongside related metabolites (cotinine, hydroxycotinine, and nicotine glucuronide) in 7 out of 120 people in both urine and plasma (Fig. 6a). Naphthalene, an industrial chemical that can also be found in mothballs, tobacco smoke, and other environmental sources, was detected as hydroxynaphthalene sulfate (naphthol sulfate) and hydroxynaphthalene glucuronide (naphthol glucuronide) in 4 out of 120 people in urine. In two out of the four individuals with naphthalene exposures detectable in urine, hydroxynapthalene sulfate was also detected in plasma (Fig. 6a). Omeprazole, an over-the-counter medication used for the treatment of heartburn, was detected alongside related metabolites (deoxyomeprazole, omeprazole sulfate, omeprazole glucuronide) in 7 out of 120 people in urine or plasma. Piperine, a chemical found in black pepper, was detected in all plasma samples. Hydroxypiperine and piperine dihydrodiol were correlated with piperine in plasma (Supplementary Fig. 4). In addition to correlations of related metabolites within a single biofluid, some compounds were correlated across plasma and urine samples (Supplementary Fig. 4). All of these chemicals were detected at the same accurate mass *m*/*z* and retention time as S9-enzyme generated metabolites. While obtaining MS^2^ spectra for each metabolite was not possible due to low abundance, at least one metabolite from each group of related xenobiotics had MS^2^ spectra that matched MS^2^ spectra from S9-enzyme generated metabolites (Fig. 6b). Thus, large-scale HRMS characterization of S9-enzymatic generated biotransformation products of diverse xenobiotics provides a strategy to identify exposures based on co-occurrence of related metabolites to complement traditional approaches to MS-based metabolites identification.

## Discussion

The purpose of this study was to develop strategies to aid the identification of low abundance xenobiotic metabolites detected by HRMS. These features, which comprise a majority of detected features, are not easily identified using only mass spectrometry-based criteria which require matching MS^1^, MS^2^, and/or MS^*n*^ spectra and retention times of unknown metabolites with those obtained from analysis of authentic standards^6,7^. Confidence in metabolite identification increases when multiple lines of evidence are provided. For example, while the use of a single accurate mass MS^1^ is not sufficient to assign a single elemental composition^23^, co-detection of naturally occurring isotopic ions and adducts increases confidence in assigning elemental compositions to detected ions. Along the same vein, incorporating biological criteria based on co-detection of related xenobiotic metabolites increases confidence in the identification of xenobiotic exposures in humans. Furthermore, co-detection of related xenobiotic metabolites in different biofluid types provides additional identification confidence for exposure assessment. Since MS^2^ and MS^*n*^ cannot always be collected from low abundance xenobiotics, generation of xenobiotic pathway metabolites using enzymatic systems enables co-detection of related xenobiotics and xenobiotic metabolites (at characteristic *m*/*z* and RT) to be used as criteria for metabolite identification. These strategies provide a complementary approach to increase the confidence of metabolite identifications, especially for low abundance xenobiotic metabolites.

Here, we demonstrate that pooled human liver S9 fraction preparations in 96-well formats provide a generalized reaction system suitable for generating Phase I and Phase II metabolites of several xenobiotics in a single experiment. We demonstrate that the addition of stable-isotope precursors to metabolite identification workflows aids structural determinations and identification of unreported metabolites. Furthermore, we show that xenobiotic metabolites produced from S9 reactions can be considered authentic metabolites for identification. Using five-minute dual chromatography HRMS methods aimed at increasing sample throughput, each 96-well experiment requires one day of duplicate sample analysis and can test eleven compounds with four-time points. Using this strategy with the assumption that each parent xenobiotic reaction generates five metabolites, fifty-five biotransformation products plus eleven parent xenobiotics could be characterized in a single day of sample analysis. Preliminary data (Supplementary Fig. 5) shows that 75% of metabolites characterized from a single chemical incubation can be captured with pooled incubation strategies. Thus, the adoption of pooled xenobiotic mixtures into experimental workflows could, in principle, increase reaction throughput to more efficiently cover exposome space. As compound libraries are screened with this approach, it will be necessary to automate data acquisition and analysis pipelines to enhance data sharing across laboratories.

While pooled human liver S9 fractions are useful models to predict primary human metabolites in vivo^14^, other biology-based systems using purified enzymes, microsomes, cell lines, primary human hepatocytes, or other cocultured cell systems^24^ could also be used for this application. Future studies could perform a systematic comparison of the performance of our S9 incubations with cell-based systems and the ability to recapitulate metabolites detected in human and mouse studies.

Some limitations to this approach require discussion. Not all xenobiotics undergo appreciable biotransformation from human enzymes. For these cases, the use of co-detection of related xenobiotics as a criterion for identification cannot be used. Furthermore, heterogeneity in xenobiotic-metabolizing enzyme expression in populations could cause differences in expression of Phase I and II metabolites that are co-detected in humans. In addition, without the use of appropriately labeled stable isotope precursors (which can be cost-prohibitive for high-throughput analysis) or use of optimized chromatographic separations (to increase analyte retention to better separate positional isomers and identify shifts in analyte polarity resulting from biotransformation), collection of MS^2^/MS^*n*^ spectra (which can identify biotransformation products based on the formation of common substructures), or additional analytic tools (ion mobility, nuclear magnetic resonance), complete characterization of stereochemistry or regiochemistry is not yet possible using mass spectrometry alone. For these cases, metabolites should be identified with broader nomenclature (e.g., hydroxybupropion versus (2S,3S)-hydroxybupropion or 4′-hydroxybupropion) without the use of positional modifications or stereochemistry, unless additional evidence is provided. Nonetheless, establishing a database of xenobiotic precursor–product relationships for identification of co-occurring related xenobiotics in samples increases identification confidence by providing orthogonal criteria for identification of suspected exposure. Existing criteria for mass spectrometry-based chemical identification confidence^6,7^ do not consider biological criteria as outlined in this article. Therefore, we have provided mass spectrometry-based criteria alongside orthogonal biological criteria for compounds identified in this work. Other aspects of translation to human analysis could be important, such as the use of serum versus plasma or the use of different anticoagulants for plasma preparation. These could impact the detection of specific chemical classes and could be evaluated in future studies.

In conclusion, the use of biologically-based enzymatic systems such as S9 fractions to generate mixtures of authentic xenobiotic metabolites for the identification of xenobiotic pathways provides a useful strategy for identifying low abundance xenobiotics detected in human samples. This approach could be adapted to work with cell-based systems and could be scaled to produce thousands of xenobiotic metabolites in a single experiment. Used alongside biological criteria based on co-detection of multiple verified xenobiotics within a pathway, adoption of a community-based xenobiotic identification strategy could be developed to address the current limitations in identifying metabolites of the tens of thousands of chemicals used in commerce^25^. Ultimately, combining the power of multiplexed enzymatic reactions of xenobiotics with computational strategies for xenobiotic pathway enrichment analysis under development for mass spectrometry data would enable higher confidence identification of unknown xenobiotic exposures.

## Methods

### Materials

Acetaminophen (APAP) (>99%), β-naphthoflavone (β-NF) (>98%), caffeine (>99%), ^13^C_3_-caffeine (>99%), nicotine (>99%), chlorzoxazone (CLZ) (98%), tolbutamide (>99%), bupropion (98%), warfarin (>98%), carvedilol (>98%), metoprolol (>98%), adenosine 3′-phosphate 5′-phosphosulfate lithium salt hydrate (PAPS) (>60%), glutathione (GSH) (98%), acetyl-coenzyme A (acetyl-CoA) (93%), and HPLC grade acetonitrile were purchased from Sigma-Aldrich (St. Louis, MO). Caffeine-d_3_ (1-methyl-d_3_) (99%) and bupropion-d_9_ (tert-butyl-d_9_) (99%) were purchased from CDN isotopes (Point-Clare, Quebec, Canada). Pazopanib, benzo[a]pyrene, and uridine diphosphate glucuronic acid (UDPGA) (>98%) were purchased from US Biological (Salem, MA). Other precursors (>95% purity) were sourced from the Spectrum Collection purchased from Microsource Discovery Systems (Gaylordsville, CT). Pooled human liver S9 fraction: mixed-gender (from 50 livers, H0640.S9) and NADPH regenerating system (K5000-10) were from Sekisui Xenotech (Kansas City, KS).

#### Xenobiotic incubations

Incubations of xenobiotics with human liver S9 fraction were carried out with minor modifications to the method of Richardson^14^. Pooled human liver S9 fractions (20 mg/ml protein) were aliquoted into 0.5 ml microcentrifuge tubes and, stored at −80 °C, and then thawed at room temperature prior to use. Stock solutions of 5 mM β-NF, tolbutamide, bupropion, pazopanib, and benzo[a]pyrene were prepared in DMSO, while 5 mM stock solutions of APAP, caffeine, nicotine, and chlorzoxazone were prepared in water. Each test compound stock solution was diluted to 0.3 mM with water prior to incubation with S9 enzymes. The NADPH regenerating system was reconstituted with the addition of 3.5 ml of water to make a final volume of 5 ml. Cofactors were combined to form a 4× cofactor stock as follows, prior to addition into the reaction mixture: 10 mM UDPGA, 2 mM GSH, 2 mg/ml PAPS, 0.1 mM acetyl-CoA, and NADPH regenerating system (1 mM NADP, 5 mM glucose-6-phosphate, 1 unit glucose-6-phosphate dehydrogenase). Reactions were carried out at 30 °C on 96-well plates (Fig. 1). S9 fraction was diluted 10-fold in water immediately before mixing with 0.2 M Tris-Cl, pH 7.5/2 mM MgCl_2_ and 0.3 mM of the xenobiotic solution in a 1:1:1 ratio (15 μL each). and incubated at 30 °C for 5 min. To start the reaction, 15 μL of 4× cofactor stock was added, and incubation was carried out at 30 °C for the indicated times. To terminate the reaction, we added a three-fold volume of acetonitrile, covered the plate with parafilm, vortexed, and froze at −20 °C to precipitate insoluble materials such as protein. After thawing and centrifugation of the incubation plate, the supernatants were transferred into polypropylene autosampler vials, which were stored at −20 °C until instrumental analysis.

### High-resolution mass spectrometry

Totally, 10 μL aliquots of sample extracts were analyzed using liquid chromatography (Dionex Ultimate 3000) and Fourier-transform HRMS (Thermo Scientific Fusion or Thermo Scientific High-field Q-exactive). The chromatography system was operated in a dual pump configuration to enable parallel analyte separation and column flushing. Sample extracts were injected and analyzed using hydrophilic interaction liquid chromatography (HILIC) with electrospray ionization (ESI) operated in positive mode and reverse phase (C18) chromatography with ESI operated in negative mode. For S9 reaction extracts, each analysis was performed using two biological replicates and a single technical replicate injection per column. For mouse and human samples, each analysis was performed using three technical replicate injections per column. Analyte separation for HILIC was accomplished using a Waters XBridge BEH Amide XP HILIC column (2.1 mm × 50 mm, 2.5 μm particle size) and eluent gradient (A = water, B = acetonitrile, C = 2% formic acid) consisting of an initial 1.5 min period of 22.5% A, 75% B, 2.5% C, followed by a linear increase to 75% A, 22.5% B, 2.5% C at 4 min and a final hold of 1 min. C18 chromatography was performed using Higgins Targa C18 2.1 mm × 50 mm, 3 μm particle size) a column with an eluent gradient (A = water, B = acetonitrile, C = 10 mM ammonium acetate) consisting of an initial 1 min period of 60% A, 35% B, 5% C, followed by a linear increase to 0% A, 95% B, 5% C at 3 min and held for the remaining 2 min. For both methods, mobile phase flow rate was held at 0.35 ml/min for the first 1 min, then increased to 0.4 ml/min for the final 4 min. The void volume elution time for each method was 18 s. The high-resolution mass spectrometer collected data at 120,000 resolution for MS^1^ data collection. Data were collected from 85 to 1275 *m*/*z* for MS^1^ analysis with additional fragmentation spectra collected for predicted xenobiotic metabolites of a test substrate using a targeted inclusion list with data-dependent MS/MS. Quality control samples consisting of pooled xenobiotic reactions were analyzed at the beginning, middle, and end of each analytical run (every twenty samples) to assess chromatographic peak quality (±8 s) and mass accuracy (observed mass ±5 ppm of expected mass) for compounds present in the pool. Additional quality assessment was performed on a compound by compound basis based upon peak quality and mass accuracy of precursor compounds. This data is available at the NIH Common Fund’s National Metabolomics Data Repository (NMDR) website, the Metabolomics Workbench, http://www.metabolomicsworkbench.org where it has been assigned project ID PR001099. The data can be accessed directly via its project 10.21228/M8N97J.

### Processing of mass spectrometry data in S9 experiments

Raw MS^1^ and MS^2^ data were processed in xCalibur QualBrowser. Expected metabolites for each xenobiotic substrate were identified using literature searches and the web-based metabolite prediction tool Biotransformer (http://biotransformer.ca)^10^. Extracted ion chromatograms were generated from accurate mass MS^1^ spectra for expected M + H for HILIC+ or M–H for C18-ions ±3 ppm and plotted using GraphPad Prism 6.0.

### Metabolite identification criteria

The present research is focused on improving capabilities for xenobiotic metabolite identification when authentic chemical standards are not available. These criteria are based on metabolomics standards initiative (MSI) minimum standards for chemical reporting^7^. MSI level 1 identification requires at least two orthogonal criteria (accurate mass and retention time or accurate mass/isotope pattern with MS/MS and retention time) to be matched from an experimental sample with an authenticated chemical standard analyzed in house. In the present study, at least three orthogonal criteria were used for metabolite identification for enzymatically generated metabolites and detected xenobiotics in experimental samples as described below. Authentication of xenobiotics was based on (1) presence of appropriate MS signals only in samples where xenobiotic was added, (2) accurate mass MS^1^ ± 3 ppm of expected masses, and MS^2^ spectra (if available) matching known or predicted patterns. Based on MSI criteria, identifications for precursor xenobiotics were considered level 1 identifications based on analysis of validated chemical standards. Enzymatically generated xenobiotic metabolites (from S9 reactions) were analyzed as crude extracts and were not additionally fractionated or purified prior to sample analysis. Identification of xenobiotic metabolites produced from S9 preparations was based on (1) presence of predicted xenobiotic metabolite only in samples where xenobiotic was added, (2) accurate mass MS^1^ ±3 ppm of expected mass for predicted enzymatic product, and (3) time-dependent increase in MS^1^ intensity only in samples where co-substrate for the enzymatic reaction was added. Based on MSI criteria, identifications for xenobiotic metabolites generated enzymatically are considered MSI level 2 (if MS/MS is available) because spectral data from enzymatically generated metabolites were not compared to purified authenticated chemical standards. Identification in clinical and experimental samples was based upon (1) detection of the expected MS^1^ mass only in the samples with documented xenobiotic exposure matching the accurate mass MS^1^ ±3 ppm of observed mass from authentic xenobiotic, (2) coelution (±5 s) with the authentic xenobiotic, and (3) co-occurrence of more than one related xenobiotic at characteristic *m*/*z* and retention time as established from analysis of S9 extracts. Mass fragmentation spectra (MS^2^) were collected when available and provided a fourth criterion for identification. Satisfying at least three orthogonal criteria as outlined here constituted level 1 confidence by orthogonal criteria (with two criteria being level 2 confidence, and only one criterion being level 3 confidence).

### Identification of unreported metabolites with stable isotopes

Caffeine, caffeine-d_3_, ^13^C_3_-caffeine, bupropion, and bupropion-d_9_ were incubated separately using the S9 fraction conditions described with samples collected at 0 and 24 h. Following instrumental analysis, data from HILIC/ESI+ analysis were extracted and aligned using mzMine2^26^. Metabolites increased at 24 h were identified using a Pearson’s correlation (*R* > 0.9 for *m*/*z* peak intensity vs. time) for labeled and unlabeled experiments. A mass difference filter (based on mass differences between expected isotope-labeled forms and native forms) was applied to identify metabolites with increases in a corresponding labeled form. Features that did not have a corresponding increase in a labeled form that co-eluted with the unlabeled form were considered nonspecific metabolites.

### Mouse samples

All animal procedures were reviewed and approved by the Institutional Animal Care and Use Committee of Emory University and complied with all relevant regulations regarding the use of research animals. Female C57BL/6J mice (aged 6–8 weeks old) from The Jackson Laboratory (Bar Harbor, ME) were housed under standard conditions and fed a normal diet (LabDiet, St. Louis, MO; chow 5001) with water ad libitum. Mice were administered a five-drug cocktail containing caffeine (5 mg/kg), bupropion (30 mg/kg), tolbutamide (5 mg/kg), bufuralol (10 mg/kg), and midazolam (3 mg/kg) in saline via oral gavage. Totally, 10 µL of blood was collected from the transected tail tips at 10, 20, 40, 60, 120, 240, 360, 480 min after drug cocktail administration into microcentrifuge tubes containing heparin 10 µL). Samples were prepared with the addition of 95 µL acetonitrile, vortexed, and centrifuged at 12,000*g* for 5 min. Samples were analyzed as described above. Mice were monitored for general health, weight loss, and anemia during drug cocktail treatments to ensure they did not reach IACUC endpoints.

### Human samples

Two sample sets were used in this study. Human samples were obtained with informed consent under IRB00090101 and IRB00007243, which were approved by the Emory University Institutional Review Board. Both study designs comply with all relevant regulations regarding the use of human study participants and were conducted in accordance to the criteria set by the Declaration of Helsinki. EDTA plasma samples (*n* = 51) from nonsmoking individuals with documented pharmaceutical use were from 11 patients participating in a clinical trial at Emory University Hospital (ClinicaTrials.gov Identifier: NCT02922816, IRB00090101). Of these 11 patients, 6 (55%) were female, 8 (73%) were white, 3 (27%) were Hispanic, and their median age was 65 years old (IQR 53–73). Repeat samples from each patient were collected on day 1, day 15, and day 36 of each study visit cycle. During the trial, each individual continued medications prescribed as part of routine clinical care. Each individual had a distinct set of medications taken as prescribed. Other demographic information on these samples was not available. The exposures investigated reflect clinical care decisions for the participants and are not related to the outcomes of the clinical trial. Urine and EDTA plasma samples from individuals with no documented pharmaceutical use (*n* = 120) were from a subset of baseline samples from adults enrolled in the Emory-Georgia Tech Centers for Health Discovery and Well-Being (CHDWB) study^27–29^, which was approved by the Emory University Institutional Review Board IRB00007243). These individuals were primarily employed by Emory University or Emory healthcare systems and recruited through the use of flyers posted around the university or by word of mouth. Participants were required to be 18 years of age or older with no acute illness or hospitalization within the previous year. Individuals were excluded by having a current diagnosis of chronic respiratory disease, chronic infection, pro-inflammatory or autoimmune disease, or a current active malignant neoplasm other than localized basal cancer of the skin. Of these 120 participants, 37 (31%) were male and 87 (69%) were female, and their median age was 53. Spectral feature tables from these studies were extracted and aligned using apLCMS^30,31^ and xMSanalyzer^32^. Correlation networks of xenobiotics were generated using xMWAS^33^ using the PLS regression function with a correlation threshold of 0.4.

### Reporting summary

Further information on research design is available in the Nature Research Reporting Summary linked to this article.

## Supplementary information

Supplementary Information

Peer Review File

Description of Additional Supplementary Files

Supplementary Data 1

Reporting Summary

## Data Availability

Source data for all figures and supplementary information are provided with this paper. The raw source data for S9 reactions generated in this study have been deposited in the Metabolomics Workbench database [https://www.metabolomicsworkbench.org] with project number PR001099 and 10.21228/M8N97J at the following link: https://metabolomicsworkbench.org/data/DRCCMetadata.php?Mode=Study&StudyID=ST001715&StudyType=MS&ResultType=5. The processed spectral data and precursor-product data are available at http://metabolomics.cloud/#/project/CIDC001. Additional data are available upon reasonable request from the corresponding authors. Source data are provided with this paper.

## Source data

Source Data
