# Supplementary material for: Large scale enzyme based xenobiotic identification for exposomics
Source: Nat Commun. 2021 Sep 14;12:5418. doi: 10.1038/s41467-021-25698-x (PMC8440538; doi:10.1038/s41467-021-25698-x)
Supplement: Supplementary file 1 — Supplementary Information [file 41467_2021_25698_MOESM1_ESM.pdf]

Supplementary information for *Large scale enzyme based xenobiotic identification for exposomics*

S1: Time-dependent formation of expected metabolites

S2: Relationship of 213.0981 *m/z* to caffeine in mice and humans

S3: Correlations of related metabolites in documented exposures

S4: Correlations of related metabolites in undocumented exposures

S5: Time-dependent formation of expected metabolites from mixtures analysis

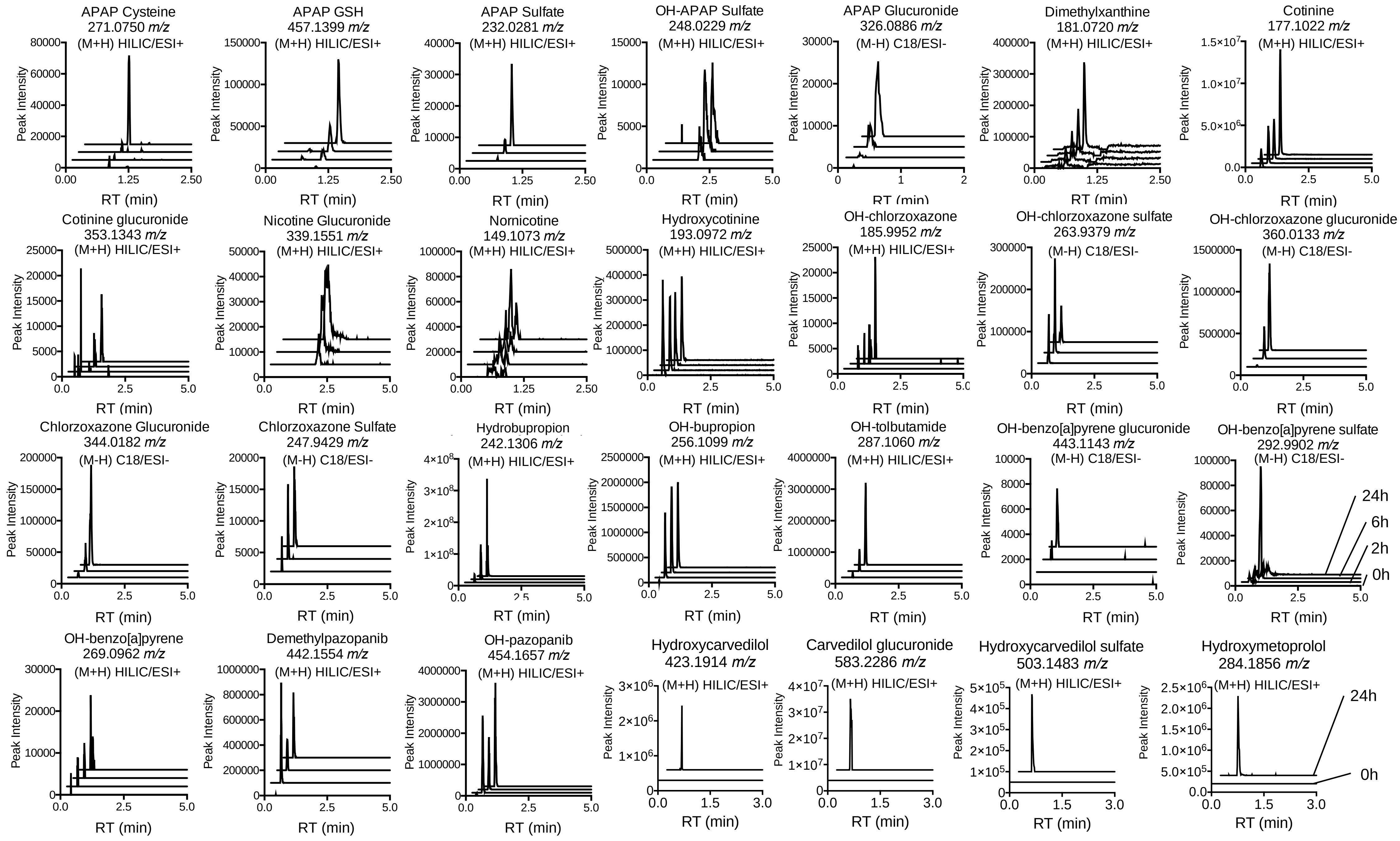

Supplementary Fig. 1. Extracted ion chromatograms for S9 enzymatic reactions showing time-dependent formation (0, 24h) of expected metabolites.

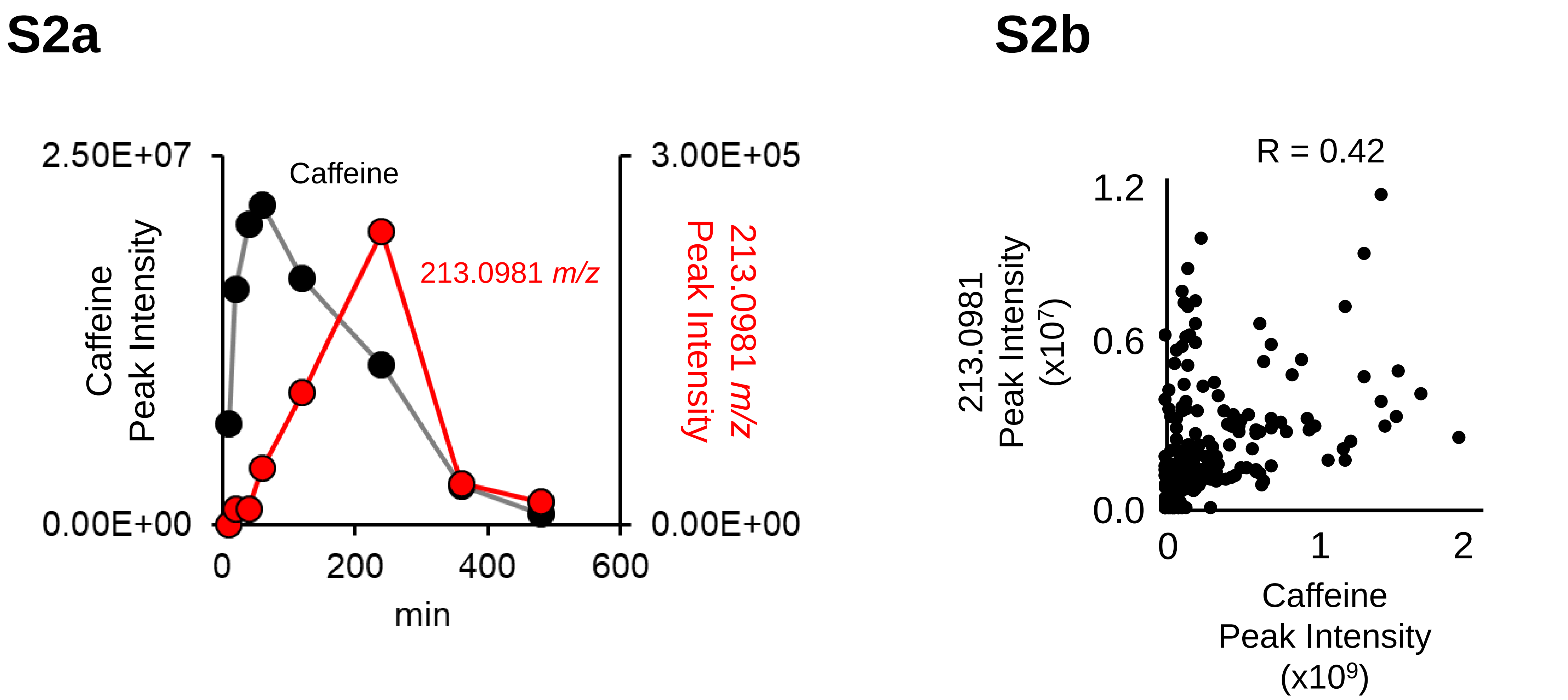

Supplementary Fig. 2. Additional data on identification of caffeine related metabolite in experimental samples

a) Pharmacokinetic profile of caffeine and previously uncharacterized caffeine-related metabolite 213.0981 m/z in mouse

b) b) Correlation of caffeine with caffeine-related metabolite 213.0981 m/z in humans (Pearson's R)

Supplementary Fig. 3.

a) Correlation-based networks of parent xenobiotics shows parent xenobiotics are correlated with expected metabolites, source fragments, adducts, and isotopes in human plasma

b) b) Co-detection of related xenobiotics in human plasma samples with documented pharmaceutical use

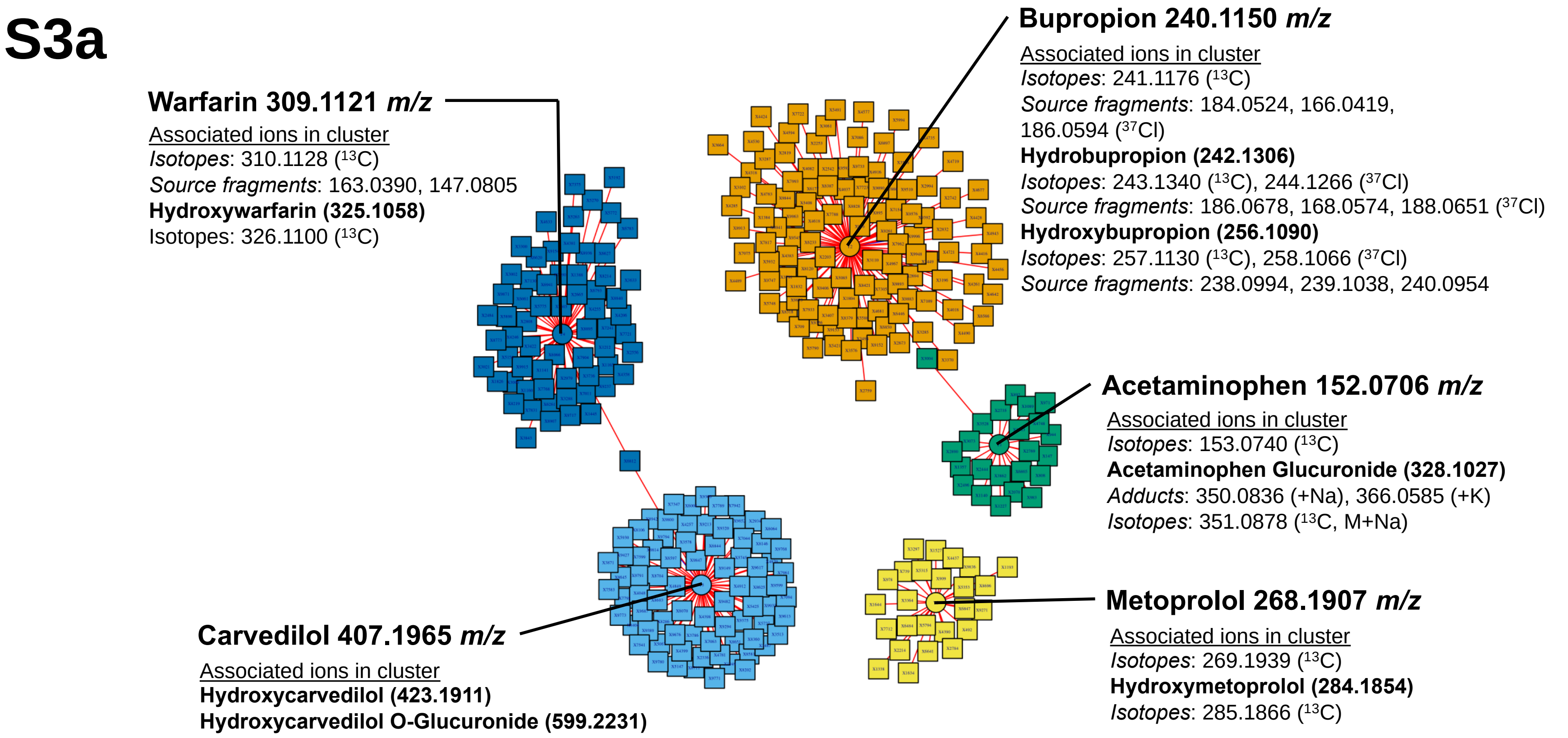

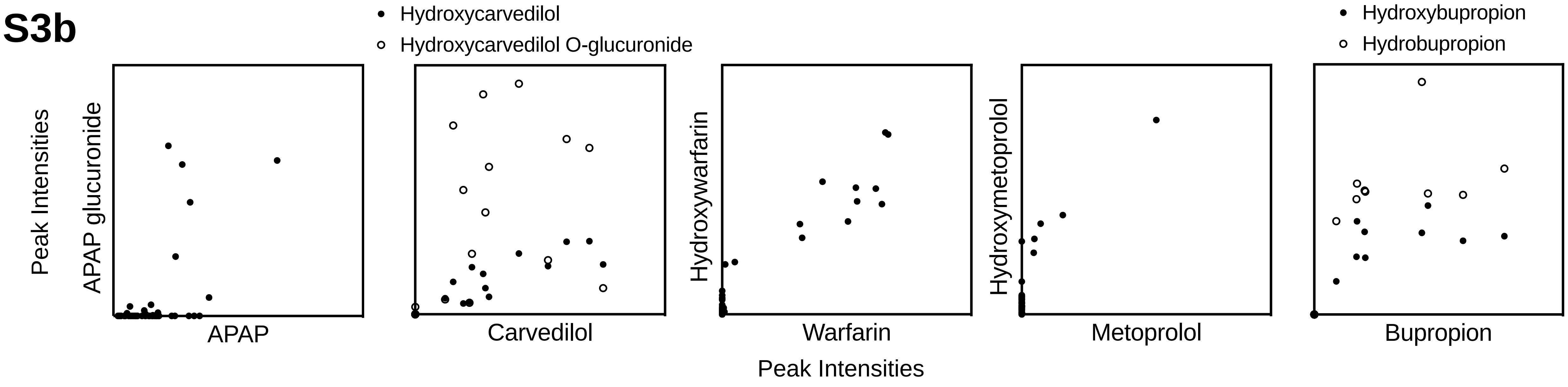

Supplementary Fig. 4.

Correlation heat maps (Pearson's R) for xenobiotic and related biotransformation products across plasma and urine samples: a. Nicotine, b. Acetaminophen, c. Ibuprofen, d. Omeprazole, e. Naphthalene, f. Piperine

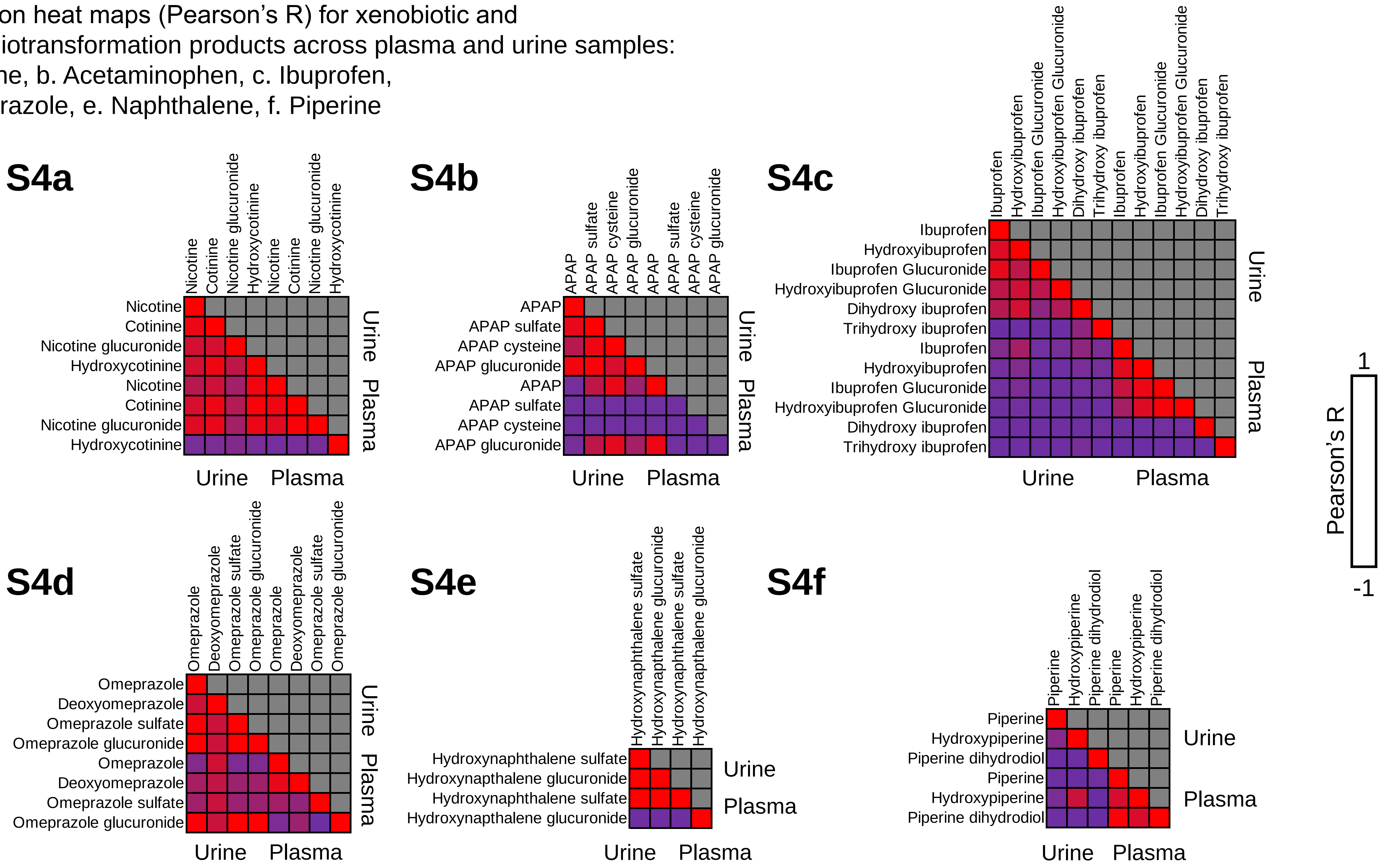

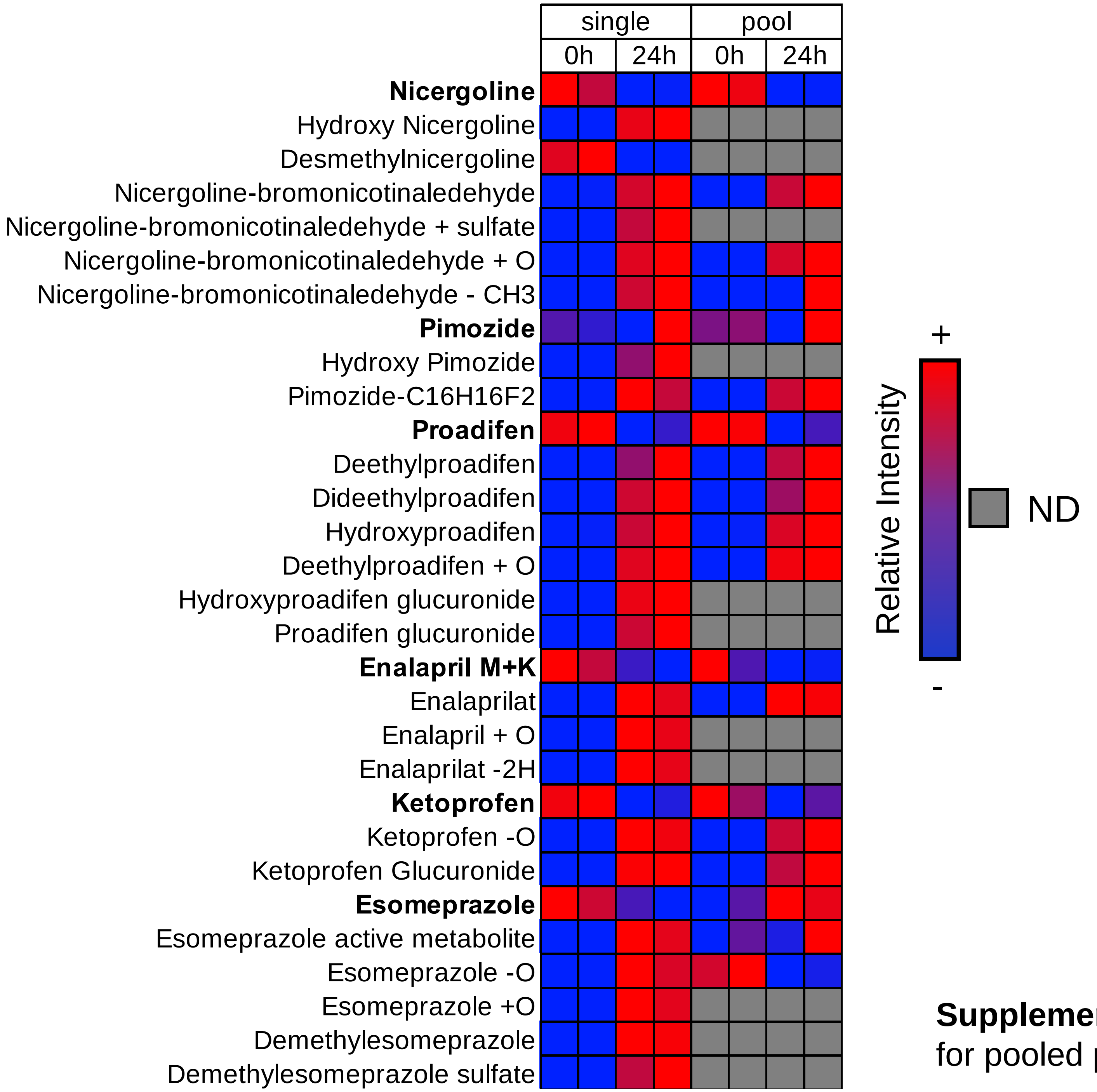

Supplementary Fig. 5. Comparison of S9 system performance for pooled precursors vs. single precursor incubations
