# Supplementary material for: Large scale enzyme based xenobiotic identification for exposomics
Source: Nat Commun. 2021 Sep 14;12:5418. doi: 10.1038/s41467-021-25698-x (PMC8440538; doi:10.1038/s41467-021-25698-x)
Supplement: Supplementary file 3 — Description of Additional Supplementary Files [file 41467_2021_25698_MOESM3_ESM.docx]

Description of Additional Supplementary Files

Title: Supplementary Data 1

Description: List of identified precursors and associated biotransformation products from S9 enzymatic reactions
