# Supplementary material for: Large scale enzyme based xenobiotic identification for exposomics
Source: Nat Commun. 2021 Sep 14;12:5418. doi: 10.1038/s41467-021-25698-x (PMC8440538; doi:10.1038/s41467-021-25698-x)
Supplement: Supplementary file 5 — Reporting Summary [file 41467_2021_25698_MOESM5_ESM.pdf]

nature research

Corresponding author(s): Edward Morgan, Dean P. Jones

Last updated by author(s): Jun 15, 2021

Reporting Summary

Nature Research wishes to improve the reproducibility of the work that we publish. This form provides structure for consistency and transparency in reporting. For further information on Nature Research policies, see our Editorial Policies and the Editorial Policy Checklist.

Statistics

For all statistical analyses, confirm that the following items are present in the figure legend, table legend, main text, or Methods section.

n/a	Confirmed	
☐	☒	The exact sample size (n) for each experimental group/condition, given as a discrete number and unit of measurement
☒	☐	A statement on whether measurements were taken from distinct samples or whether the same sample was measured repeatedly
☒	☐	The statistical test(s) used AND whether they are one- or two-sided<br>*Only common tests should be described solely by name; describe more complex techniques in the Methods section.*
☒	☐	A description of all covariates tested
☒	☐	A description of any assumptions or corrections, such as tests of normality and adjustment for multiple comparisons
☒	☐	A full description of the statistical parameters including central tendency (e.g. means) or other basic estimates (e.g. regression coefficient) AND variation (e.g. standard deviation) or associated estimates of uncertainty (e.g. confidence intervals)
☒	☐	For null hypothesis testing, the test statistic (e.g. F, t, r) with confidence intervals, effect sizes, degrees of freedom and P value noted<br>*Give P values as exact values whenever suitable.*
☒	☐	For Bayesian analysis, information on the choice of priors and Markov chain Monte Carlo settings
☒	☐	For hierarchical and complex designs, identification of the appropriate level for tests and full reporting of outcomes
☐	☒	Estimates of effect sizes (e.g. Cohen's d, Pearson's r), indicating how they were calculated

Our web collection on statistics for biologists contains articles on many of the points above.

Software and code

Policy information about availability of computer code

Data collection	Thermo Scientific XCalibur for Mass spectrometry data collection
Data analysis	Graphpad Prism 6.0, Biotransformer, apLCMS, xMSanalyzer, mzMine 2.0

For manuscripts utilizing custom algorithms or software that are central to the research but not yet described in published literature, software must be made available to editors and reviewers. We strongly encourage code deposition in a community repository (e.g. GitHub). See the Nature Research guidelines for submitting code & software for further information.

Data

Policy information about availability of data

All manuscripts must include a data availability statement. This statement should provide the following information, where applicable:

- Accession codes, unique identifiers, or web links for publicly available datasets
- A list of figures that have associated raw data
- A description of any restrictions on data availability

Raw data for S9 reactions are available at Metabolomics Workbench DOI: 10.21228/M8N97J. Associated chromatograms and spectral information is available at http://metabolomics.cloud/$/project/CIDC001. All other data are available upon reasonable request.

Field-specific reporting

Life sciences study design

All studies must disclose on these points even when the disclosure is negative.

Sample size	This study used existing archival human data collected for non-targeted metabolomics analyses. 120 samples was considered suitable to test for detection of common drug, environmental, and dietary exposures (omeprazole, cotinine/nicotine, naphthalene, piperine). Guidance from a CDC exposure assessment laboratory for CLIA states that a minimum of 120 individual be used for reference ranges: https://www.aphl.org/aboutAPHL/publications/Documents/EH_2013Dec_CLIA-Compliant-LRN-C-Method-Validation-Template.pdf Samples collected from clinical trial participants (n = 11, with repeat measures per individual) were used to evaluate methods for detection of drug metabolites from documented drug exposures. Samples from 11 individuals with documented use of 5 target drugs were available and all 11 were studied.
Data exclusions	No data were excluded from the study.
Replication	For the paired urine and plasma samples from the same individual, co-detection of related metabolites between two sample matrices often provides relevant replication of exposure-related biomarkers. For clinical trail participants, we have repeat samples from the same individual where identified exposures were replicated in repeat samples. We have validated the workflow for identification of metabolites in animal and human samples. All samples (clinical trial participants, CHDWB cohort) were analyzed using three technical replicates per sample. Chemical detection is replicated across all three technical replicates.
Randomization	Human samples were randomized prior to sample analysis.
Blinding	For samples with documented drug exposures, investigators had access to electronic health records containing drug use which was used to validate detection of known drug exposures. For CHDWB cohort, investigators were blinded to potential environmental, diet, or drug exposures.

Reporting for specific materials, systems and methods

We require information from authors about some types of materials, experimental systems and methods used in many studies. Here, indicate whether each material, system or method listed is relevant to your study. If you are not sure if a list item applies to your research, read the appropriate section before selecting a response.

Materials & experimental systems

n/a	Involved in the study	
☒	☐	Antibodies
☒	☐	Eukaryotic cell lines
☒	☐	Palaeontology and archaeology
☒	☐	Animals and other organisms
☐	☒	Human research participants
☒	☐	Clinical data
☒	☐	Dual use research of concern

Methods

n/a	Involved in the study	
☒	☐	ChIP-seq
☒	☐	Flow cytometry
☒	☐	MRI-based neuroimaging

Human research participants

Policy information about studies involving human research participants

Population characteristics	The present study was to develop and validate methods for identification of xenobiotic exposures in humans. Samples from from clinical trial participants (n = 11) being treated at Emory University Hospital were composed of 55% female, 73% white, and 27% hispanic. Median age was 65 (IQR 53-73). Samples from CHDWB cohort (n = 120) were volunteers (mostly Emory University staff) with 31% male and 69% female. Median age was 53.
Recruitment	CHDWB cohort were healthy volunteers from Emory University recruited through flyers and electronic mail. These individuals may not be representative of a general population.
Ethics oversight	Emory University IRB00090101, IRB00007243

Note that full information on the approval of the study protocol must also be provided in the manuscript.
