# Supplementary material for: Large scale enzyme based xenobiotic identification for exposomics
Source: Nat Commun. 2021 Sep 14;12:5418. doi: 10.1038/s41467-021-25698-x (PMC8440538; doi:10.1038/s41467-021-25698-x)
Supplement: Supplementary file 2 — Peer Review File [file 41467_2021_25698_MOESM2_ESM.pdf]

nature portfolio

Peer Review File

Large scale enzyme based xenobiotic identification for exposomics

Open Access This file is licensed under a Creative Commons Attribution 4.0 International License, which permits use, sharing, adaptation, distribution and reproduction in any medium or format, as long as you give appropriate credit to the original author(s) and the source, provide a link to the Creative Commons license, and indicate if changes were made. In the cases where the authors are anonymous, such as is the case for the reports of anonymous peer reviewers, author attribution should be to 'Anonymous Referee' followed by a clear attribution to the source work. The images or other third party material in this file are included in the article's Creative Commons license, unless indicated otherwise in a credit line to the material. If material is not included in the article's Creative Commons license and your intended use is not permitted by statutory regulation or exceeds the permitted use, you will need to obtain permission directly from the copyright holder. To view a copy of this license, visit http://creativecommons.org/licenses/by/4.0/.

Reviewers' Comments:

Reviewer #1:
Remarks to the Author:
The measurement of the internal exposome in human studies faces major challenges due to the diversity of compounds, their low concentrations and difficulties in obtaining high quality MS/MS data, their extensive biotransformation in the organism, and the lack of authentic chemical standards. This very much limits progress in this field of research. To circumvent these limitations, the authors generate metabolites of xenobiotics by reaction with a commercial pooled human S9 fraction, build a retention time/accurate mass library of xenobiotic metabolites, use metabolite/metabolite correlations and mass differences, compare drug metabolites formed in vitro with those observed in patients treated with the same drug. They also describe the use of labelled compounds to trace xenobiotic metabolites.
These approaches will help in the annotation of the exposome by producing authentic chemical standards, often not available from chemical companies. These approaches have been used earlier to identify metabolites from drugs, contaminants, or foods although not on a large scale. This study, nicely written and presented, has the merit to draw the attention of the exposome community to these techniques.
Methods are applied to a small panel of about 15 xenobiotics in a proof of principle study. Further work will be needed 'to analyze the thousands of xenobiotic metabolites in a single experiment' (line 328).
These approaches, although all valid and useful, may not be sufficient to identify with sufficient certainty unknown xenobiotic metabolites. For example, in Figure 4, a new metabolite of caffeine is described. This metabolite differs from 1,3,7-trimethyluric acid, a well-known metabolite of caffeine, by the reduction of the carbonyl group on carbon 6. This new metabolite has apparently never been described for compounds as widely studied as caffeine or xanthine-related compounds. The spectra given in figure 4 should be commented and more data (e.g. NMR) will likely be needed to establish the proposed structure with sufficient confidence.
Also, this metabolite shown in figure 4 is not a good example to demonstrate the usefulness of labelled compounds in establishing a new chemical structure, or the spectra in Figure 4d-f should be commented to make this clearer.

In total, the approaches presented in this paper will be important for the annotation of the exposome. However, it should also make clear in the discussion that it may not be sufficient to identify many constituents of the exposome. Other methodological developments will also be equally important and needed like collection of high-quality MS/MS spectra for all compounds, including those present at low concentrations.

Some additional comments:
Line 72: Ref #5 is not the right reference. The following reference should be cited instead: Rappaport, S. M.; Barupal, D. K.; Wishart, D.; Vineis, P.; Scalbert, A., The blood exposome and its role in discovering causes of disease. Environ. Health Perspect. 2014, 122 (8), 769-74.
Lines 73-77: These few lines appear very specific for the introduction and are not needed here.
Lines 139-140: Ion dissociation spectra: Do the authors mean mass fragmentation spectra?
Line 143: Title of supplemental table 1 missing.
Line 171, Figure 3f: These spectra should be commented. It is not obvious otherwise to draw any conclusions out of them.
Line 234: Supplemental figure 2 is not useful as the ions cannot be identified (not readable on the figure).

Reviewer #2:
Remarks to the Author:
Developing a high-throughput format for S9 incubations to produce biotransformation products of diverse xenobiotics is a good idea (although routinely done for single xenobiotics) and well aligned with the concept of exposomics; so this is interesting work. My main critique is that showing the concept for nine well-known xenobiotics is not omic-scale and not what one would expect from some of the key researchers in exposomics. If the authors can demonstrate that this works for

100+ xenobiotics (incl so far less investigated) and demonstrate the impact in different matrices and cohort samples (particularly urine, if focusing on glucuronides and sulfates) it would be a major break-through and really move the field forward. Since this is named as high-throughput, it should be feasible to be done relatively easily.

o Minor point: Why use EDTA-plasma? Experiences with EDTA in HRMS is mixed and generally heparin plasma is often recommended for untargeted HRMS.

o Showing the tentative identification of new metabolites by stable isotopes was demonstrated using caffeine. While interesting that there are still unknowns for such a well-investigated molecule, it would be better to show (additionally) new metabolites of toxicants that do not occur at such high concentrations in real samples.

o What I miss (and think would be essential to make this paper a milestone and justify Nat Comm) is the large-scale application of the approach to food and environmental-related exposures (for many there is no data on phase I and II metabolism available at all). Identifying a high number of known and unknown biotransformation products and feeding the MS2 spectra into the diverse metabolome/exposome databases would be great and really showcase the vast potential of the approach.

o The 'Identification of xenobiotics in vivo from unknown exposures' part is too weak for my understanding. Showing tobacco smoke exposure is too limited in scope for true exposome work. What else was found in the cohort and how does this compare to other published exposomic screening work? I am also missing more information on the cohort itself in the main manuscript.

o The authors state the S9 fractions are better suited than microsomes or liver cells. While I tend to agree it would be nice to see the underlying data (if existing). We typically test all three as the metabolite patterns can be very different. To show the broad applicability of the novel workflow this would certainly further strengthen the paper.

o 'Adoption of pooled xenobiotic mixtures into experimental workflows could, in principle, increase reaction throughput to more efficiently cover exposome space.' It would be nice to see a proof-of-principle example to prove this capacity. Again, this would strengthen your point that this approach can be a game changer in exposomics.

o Figures are high quality but could be even more optimized to be more 'Nature style'

Reviewer #3:
Remarks to the Author:
The paper 'Large-scale, enzyme-based xenobiotic identification for exposomics' by Liu et al. is a nice contribution in the expanding field of exposome research. To make it even more valuable for the community and beyond, I recommend to expand the selection of xenobiotics and demonstrate a broader applicability. Please find some detailed comments below.

Line 65: 'high-resolution metabolomics (HRM)': This term is not used by the community and should be replaced by high-resolution mass spectrometry (HRMS).

Line 76: '…and routine analyses provide relative quantification of over 35,000 accurate mass spectral features'. To me this number seems high for biological samples measured on a Thermo HRMS instrument if the deconvolution parameters are chosen correctly. We typically see 5,000-10,000 features in one analytical run of 20-30 min.

Line 98: '…to generate metabolites from a panel of xenobiotics with known biotransformation products.' It would be important to also test this with model xenobiotics where basically nothing is known about biotransformation as proof-of-principle

Line 102: '… and we apply this principle to identify undocumented environmental exposures.' I

assume this statement refers to the section ‘Identification of xenobiotics in vivo from unknown exposures’ starting at line 252. However, the presented data on a single tobacco related metabolite did not convince me. Since this aspect of the paper is important for future application of this work, it should clearly show how it can deliver results regarding unknown exposures. Since this is exposomics work it should strive to report a multitude of different exposure classes.

Figure 1: The caption of Figure 1 does not explain what ‘C1, C2, C3 etc’ stand for. I assume it should be the individual xenobiotics? Also the two ‘pool mixes’ should be explained.

Line 133: ‘…we tested 9 xenobiotics with well characterized phase I and phase II metabolism’. I have two comments: (1) To be omics-scale, nine model substances are not sufficient. (2) Most of these xenobiotics are commonly used drugs with well-characterized pharmacokinetics. Of course the ‘exposome’ includes drugs, however, I would clearly like to see a broader selection of xenobiotics including those most important in environmental health (pesticides, plasticizers, PFAS, food contaminants, plant toxins, endocrine disrupters etc.). If at least one model xenobiotics from all these diverse classes are tested by the novel biotransformation approach, I believe this work could have a very high impact.

Figure 2: I like the content of this illustration but was wondering about the early elution of all metabolites (retention times < 1 min). What is the void volume of the used chromatographic method? It is not clear which method (RP or HILIC) was used for the specific molecules; this should be stated somewhere.

The reported peak intensities in Figures 2 and 3 are pretty high. Could you also show chromatograms of some low-abundance biotransformation products that are in the range of 10e3? Often these are the most interesting metabolic products.

In addition, I miss crucial methodological information. E.g. the MS model or the injection volume are not stated and it is unclear what kind of QC measurements were performed to ensure proper performance of the instrumental platform (for a high-throughput pipeline long-term performance proof is even more important but I could not find it in the main manuscript or the SI). It is not clear if the reported data is in line with the Metabolomics Standard Initiative (MSI) or similar initiatives promoting reproducibility and benchmarking.

In the spirit of open science I would like to see all raw and meta data provided via a mass spec repository.

REVIEWER COMMENTS

Reviewer #1 (Remarks to the Author):

The measurement of the internal exposome in human studies faces major challenges due to the diversity of compounds, their low concentrations and difficulties in obtaining high quality MS/MS data, their extensive biotransformation in the organism, and the lack of authentic chemical standards. This very much limits progress in this field of research. To circumvent these limitations, the authors generate metabolites of xenobiotics by reaction with a commercial pooled human S9 fraction, build a retention time/accurate mass library of xenobiotic metabolites, use metabolite/metabolite correlations and mass differences, compare drug metabolites formed in vitro with those observed in patients treated with the same drug. They also describe the use of labelled compounds to trace xenobiotic metabolites.
These approaches will help in the annotation of the exposome by producing authentic chemical standards, often not available from chemical companies. These approaches have been used earlier to identify metabolites from drugs, contaminants, or foods although not on a large scale. This study, nicely written and presented, has the merit to draw the attention of the exposome community to these techniques.

Methods are applied to a small panel of about 15 xenobiotics in a proof of principle study. Further work will be needed 'to analyze the thousands of xenobiotic metabolites in a single experiment' (line 328). These approaches, although all valid and useful, may not be sufficient to identify with sufficient certainty unknown xenobiotic metabolites. For example, in Figure 4, a new metabolite of caffeine is described. This metabolite differs from 1,3,7-trimethyluric acid, a well-known metabolite of caffeine, by the reduction of the carbonyl group on carbon 6. This new metabolite has apparently never been described for compounds as widely studied as caffeine or xanthine-related compounds. The spectra given in figure 4 should be commented and more data (e.g. NMR) will likely be needed to establish the proposed structure with sufficient confidence.

We have expanded the list beyond the original 15 to 140 compounds. From the 140 parent compounds, an additional 397 metabolites were identified and characterized on our HRMS platform.

Also, this metabolite shown in figure 4 is not a good example to demonstrate the usefulness of labelled compounds in establishing a new chemical structure, or the spectra in Figure 4d-f should be commented to make this clearer.

We have added additional information to the legend of figure 4 to assist with the interpretation of the MS/MS spectra.

In total, the approaches presented in this paper will be important for the annotation of the exposome. However, it should also make clear in the discussion that it may not be sufficient to identify many constituents of the exposome. Other methodological developments will also be equally important and needed like collection of high-quality MS/MS spectra for all compounds, including those present at low concentrations.

We have added additional clarification to the discussion (see page 16, lines 331-336).

Some additional comments:
Line 72: Ref #5 is not the right reference. The following reference should be cited instead: Rappaport, S. M.; Barupal, D. K.; Wishart, D.; Vineis, P.; Scalbert, A., The blood exposome and its role in discovering causes of disease. Environ. Health Perspect. 2014, 122 (8), 769-74.

We have fixed this (line 73, reference 5).

Lines 73-77: These few lines appear very specific for the introduction and are not needed here.

We have removed those lines from the introduction.

Lines 139-140: Ion dissociation spectra: Do the authors mean mass fragmentation spectra?

We have made this change.

Line 143: Title of supplemental table 1 missing.

We have added a title to the supplemental table.

Line 171, Figure 3f: These spectra should be commented. It is not obvious otherwise to draw any conclusions out of them.

We have added additional interpretation to the figure.

Line 234: Supplemental figure 2 is not useful as the ions cannot be identified (not readable on the figure).

We have added additional clarification to the list of masses contained in each cluster.

Reviewer #2 (Remarks to the Author):

Developing a high-throughput format for S9 incubations to produce biotransformation products of diverse xenobiotics is a good idea (although routinely done for single xenobiotics) and well aligned with the concept of exposomics; so this is interesting work. My main critique is that showing the concept for nine well-known xenobiotics is not omic-scale and not what one would expect from some of the key researchers in exposomics. If the authors can demonstrate that this works for 100+ xenobiotics (incl so far less investigated) and demonstrate the impact in different matrices and cohort samples (particularly urine, if focusing on glucuronides and sulfates) it would be a major break-through and really move the field forward. Since this is named as high-throughput, it should be feasible to be done relatively easily.

We have increased the number of xenobiotic compounds screened to 140 and now include this information in the manuscript/supplemental table. We have also categorized chemicals as dietary, environmental, or pharmaceuticals. We have also extended the application for identification of undocumented exposures in 120 paired urine and plasma samples and demonstrated confident identification of diverse xenobiotic classes in human samples (updated Figure 6).

o Minor point: Why use EDTA-plasma? Experiences with EDTA in HRMS is mixed and generally heparin plasma is often recommended for untargeted HRMS.

We have added a comment to the discussion (line 340-342).

o Showing the tentative identification of new metabolites by stable isotopes was demonstrated using caffeine. While interesting that there are still unknowns for such a well-investigated molecule, it would be better to show (additionally) new metabolites of toxicants that do not occur at such high concentrations in real samples.

The new updated Figure 6 contains metabolites that meet this criteria.

o What I miss (and think would be essential to make this paper a milestone and justify Nat Comm) is the large-scale application of the approach to food and environmental-related exposures (for many there is no data on phase I and II metabolism available at all). Identifying a high number of known and unknown biotransformation products and feeding the MS2 spectra into the diverse metabolome/exposome databases would be great and really showcase the vast potential of the approach.

We have extended the original application for identification of nicotine to confidently identify others undocumented xenobiotic exposures from naphthalene (moth balls), over the counter drugs (omeprazole), and dietary-related compounds such as piperine (black pepper) (Figure 6, lines 312-339).

o The 'Identification of xenobiotics in vivo from unknown exposures' part is too weak for my understanding. Showing tobacco smoke exposure is too limited in scope for true exposome work. What else was found in the cohort and how does this compare to other published exposomic screening work? I am also missing more information on the cohort itself in the main manuscript.

As indicated above, we have now identified other xenobiotic metabolites (Figure 6, lines 259-270). We have added additional cohort information into the methods section and have included references for the CHDWB study in the methods section.

o The authors state the S9 fractions are better suited than microsomes or liver cells. While I tend to agree it would be nice to see the underlying data (if existing). We typically test all three as the metabolite patterns can be very different. To show the broad applicability of the novel workflow this would certainly further strengthen the paper.

As now clarified in line 105-106, we based our selection upon existing literature (Reference 14). We have not compared microsomes and S9s on this platform and have added a relevant comment concerning potential utility in the Discussion (line 321-326).

o 'Adoption of pooled xenobiotic mixtures into experimental workflows could, in principle, increase reaction throughput to more efficiently cover exposome space.' It would be nice to see a proof-of-principle example to prove this capacity. Again, this would strengthen your point that this approach can be a game changer in exposomics.

We have added additional data to supplemental figure 3 to show that this is possible and added additional text in lines 314-316.

o Figures are high quality but could be even more optimized to be more 'Nature style'

We have updated figures 1, 3, and 6.

Reviewer #3 (Remarks to the Author):

The paper 'Large-scale, enzyme-based xenobiotic identification for exposomics' by Liu et al. is a nice contribution in the expanding field of exposome research. To make it even more valuable for the community and beyond, I recommend to expand the selection of xenobiotics and demonstrate a broader applicability. Please find some detailed comments below.

Line 65: 'high-resolution metabolomics (HRM)': This term is not used by the community and should be replaced by high-resolution mass spectrometry (HRMS).

We have made this change.

Line 76: '…and routine analyses provide relative quantification of over 35,000 accurate mass spectral features'. To me this number seems high for biological samples measured on a Thermo HRMS instrument if the deconvolution parameters are chosen correctly. We typically see 5,000-10,000 features in one analytical run of 20-30 min.

We have removed those lines from the manuscript.

Line 98: '…to generate metabolites from a panel of xenobiotics with known biotransformation products.' It would be important to also test this with model xenobiotics where basically nothing is known about biotransformation as proof-of-principle.

We have increased the number of xenobiotic compounds to 140 and these include some that are not well characterized (piperine, kojic acid, tribufos, dichlorobenzidine, 1,2-diphenylhydrazine, others).

Line 102: '… and we apply this principle to identify undocumented environmental exposures.' I assume this statement refers to the section 'Identification of xenobiotics in vivo from unknown exposures' starting at line 252. However, the presented data on a single tobacco related metabolite did not convince me. Since this aspect of the paper is important for future application of this work, it should clearly show how it can deliver results regarding unknown exposures. Since this is exposomics work it should strive to report a multitude of different exposure classes.

As indicated above, we have expanded the list of compounds and included different chemical categories (updated Figure 6, supplemental metabolite table).

Figure 1: The caption of Figure 1 does not explain what 'C1, C2, C3 etc' stand for. I assume it should be the individual xenobiotics? Also the two 'pool mixes' should be explained.

We have revised the figure to clarify this point and removed the pool mixes from the figure.

Line 133: '…we tested 9 xenobiotics with well characterized phase I and phase II metabolism'. I have two comments: (1) To be omics-scale, nine model substances are not sufficient. (2) Most of these xenobiotics are commonly used drugs with well-characterized pharmacokinetics. Of course the 'exposome' includes drugs, however, I would clearly like to see a broader selection of xenobiotics including those most important in environmental health (pesticides, plasticizers, PFAS, food contaminants, plant toxins, endocrine disrupters etc.). If at least one model xenobiotics from all these diverse classes are tested by the novel biotransformation approach, I believe this work could have a very high impact.

We have increased the number of xenobiotic compounds screened to 140 and now include this information in the manuscript/supplemental table. We have also categorized chemicals as dietary, environmental, or pharmaceuticals. We have also extended the application for identification of undocumented exposures in 120 paired urine and plasma samples and demonstrated confident identification of diverse xenobiotic classes in human samples (updated Figure 6).

Figure 2: I like the content of this illustration but was wondering about the early elution of all metabolites (retention times < 1 min). What is the void volume of the used chromatographic method? It is not clear which method (RP or HILIC) was used for the specific molecules; this should be stated somewhere.

We have revised the figure to include the analytic platform used (with void volumes included in the methods section line 413). The method used for detection of each metabolite is provided in the supplemental table containing all the identified and characterized metabolites.

The reported peak intensities in Figures 2 and 3 are pretty high. Could you also show chromatograms of some low-abundance biotransformation products that are in the range of 10e3? Often these are the most interesting metabolic products.

Some low abundance metabolites generated from *in vitro* reactions are shown in supplemental files (hydroxybenzopyrene glucuronide and others). Extracted ion chromatograms are available on the supplemental website.

In addition, I miss crucial methodological information. E.g. the MS model or the injection volume are not stated and it is unclear what kind of QC measurements were performed to ensure proper performance of the instrumental platform (for a high-throughput pipeline long-term performance proof is even more important but I could not find it in the main manuscript or the SI). It is not clear if the reported data is in line with the Metabolomics Standard Initiative (MSI) or similar initiatives promoting reproducibility and benchmarking.

We have updated the methods section to include this information (line 417-420). Metabolite ID criteria were based on MSI criteria and additional language has been added to methods (line 429-443).

In the spirit of open science I would like to see all raw and meta data provided via a mass spec repository.

We have uploaded all raw files to Metabolomics Workbench (ST001715, doi: 10.21228/M8N97J) and created a supplemental website containing reaction data.

Reviewers' Comments:

Reviewer #1:
Remarks to the Author:
All corrections were made as suggested by this reviewer.
However, the following corrections would still be needed before publication:

Page 7, lines 193-4. Names of metabolites should be corrected throughout the text and figures as follows:
- 2,3-hydroxybupropion should be (2S,3S)-Hydroxybupropion.
- 4-hydroxybupropion should be 4'-hydroxybupropion.

In figure 3 legend, some hyphens are missing in names of some chemicals.

Page 9, lines 247-249: The approach to remove false positive by testing the reaction on labelled compounds is very good. However, what about compounds that are not available as labelled compounds? What would then be the approach to properly filter the relevant peaks? In addition, the use of labelled compounds for large number of compounds will result in high costs, and this should be mentioned as part of the limitations of the approach (lines 414-426).

Page 13, line 346: Piperine is not found in Supplemental Figure 3. In Supplemental figure 3b, the nature of the biospecimens should be indicated.

Supplemental figures: Legend and figure would best appear on the same page. Check on all supplemental figure that color code appears properly (missing in suppl figure 4 and 5).

Reviewer #2:
Remarks to the Author:
The authors have responded to all of my concerns satisfactorily.

Reviewer #3:
Remarks to the Author:
The authors did a great job in revising the manuscript and providing a lot of relevant additional data. It is especially appreciated that the panel of xenobiotics was significantly increased (to 140) and that all the raw data is provided to the community now. I predict that this work will be a nice resource for others which will further enhance its impact on the expanding exposome community.

I just have some minor comments left:

Line 159: ‚It is important to note that not all metabolites were not detected at sufficient levels to collect mass fragmentation spectra�good. One ‚not‛ too much

Line 339: ‚Naphthalene, the main ingredient in moth balls, was detected as hydroxynaphthalene sulfate and hydroxynaphthalene glucuronide‛. In the opinion of this reviewer hydroxynaphthalene is mostly referred to as ‚naphtol' in the literature and main exposure is not related to moth balls but several other routes.

I like Figure 6 showing paired urine and plasma samples for multiple exposures.

The chromatographic methods do not seem well-optimized for retaining highly polar metabolic products to me as most identified metabolites elute very early (see Table S1). I do not understand why the RP method starts at 60% eluent A. This is not common and I could not find a reason/justification for that in the manuscript. Why not starting at 10% A and employing a steeper gradient? Would not increase the overall run time but better retain most of the metabolites. I do not doubt the results presented (as you have multiple layers to support your putative

identifications) but to me the chromatographic method just doesn’t look very elegant. Maybe a sentence could be added to highlight future optimization potential?

Line 531: ‚Quality control samples consisting of pooled xenobiotic reactions were analyzed at the beginning, middle, and end of each analytical run.‘ This is not sufficiently detailed (the analytical run could be either 20 injections or 200 e.g.). What was the interval? In HRMS it is generally accepted that about very 5th injection should be a pooled QC. I also miss information how the pooled QC was used to verify robust analytical performance.

Supplemental Table 1 - List of identified metabolites is very comprehensive and useful. I just would like to see information on identification level and a unique identifier (e.g. PubChem ID, ChEBI) added to help secondary use of fellow researcher using cheminformatics. This is crucial as most identifications are not at level 1 (as no reference standards are available) and HRMS non-experts may have issues interpreting the data correctly.

Some specifics I noted looking at the SI:

Hydroxywarfarin elutes at the same time (26 s) as the native metabolite. Being more polar it should have more retention on a HILIC column. Any idea why not?

Hydroxynapthalene – not found in neg ESI mode; usually it ionizes better in ESI- than ESI+

Figure S1: It is not clear what ion is depicted. Benzo[a]pyrene is given at m/z 253.1012 while the two hydroxylated metabolites are at m/z 254.0955 and 269.0962? I think there is a mistake; it is also not in line with Table S1. The m/z ratios and retention times do not match.

REVIEWER COMMENTS

Reviewer #1 (Remarks to the Author):

All corrections were made as suggested by this reviewer.
However, the following corrections would still be needed before publication:

Page 7, lines 193-4. Names of metabolites should be corrected throughout the text and figures as follows:
- 2,3-hydroxybupropion should be (2S,3S)-Hydroxybupropion.
- 4-hydroxybupropion should be 4'-hydroxybupropion.

In figure 3 legend, some hyphens are missing in names of some chemicals.

We have made these changes, updated the names of the chemicals in figure 3 and also made these changes to the figure 3 legend.

Page 9, lines 247-249: The approach to remove false positive by testing the reaction on labelled compounds is very good. However, what about compounds that are not available as labelled compounds? What would then be the approach to properly filter the relevant peaks? In addition, the use of labelled compounds for large number of compounds will result in high costs, and this should be mentioned as part of the limitations of the approach (lines 414-426).

We have added a line to the discussion (line 346-347) to address the cost of using stable isotope labels for metabolite ID and another comment (line 348-349) to address the approach to identify compounds without use of labeled precursors.

Page 13, line 346: Piperine is not found in Supplemental Figure 3. In Supplemental figure 3b, the nature of the biospecimens should be indicated.

Please see line 281-283 where we have corrected this to Supplemental Fig. 4. We have also updated the legend of Supplemental Figure 3 to clarify the nature of the biospecimens in for that figure.

Supplemental figures: Legend and figure would best appear on the same page. Check on all supplemental figure that color code appears properly (missing in suppl figure 4 and 5).

We have added additional formatting and moved some legends into the same page as the figure.

Reviewer #2 (Remarks to the Author):

The authors have responded to all of my concerns satisfactorily.

Reviewer #3 (Remarks to the Author):

The authors did a great job in revising the manuscript and providing a lot of relevant additional data. It is especially appreciated that the panel of xenobiotics was significantly increased (to 140) and that all the raw data is provided to the community now. I predict that this work will be a nice resource for others which will further enhance its impact on the expanding exposome community.

I just have some minor comments left:

Line 159: ‚It is important to note that not all metabolites were not detected at sufficient levels to collect mass fragmentation spectra‘. One ‚not‘ too much

This has been corrected. Please see line 140-142.

Line 339: ‚Naphthalene, the main ingredient in moth balls, was detected as hydroxynaphthalene sulfate and hydroxynaphthalene glucuronide‘. In the opinion of this reviewer hydroxynaphthalene is mostly referred to as ‚naphtol' in the literature and main exposure is not related to moth balls but several other routes.

We have altered the text (Line 264-265).

I like Figure 6 showing paired urine and plasma samples for multiple exposures.

The chromatographic methods do not seem well-optimized for retaining highly polar metabolic products to me as most identified metabolites elute very early (see Table S1). I do not understand why the RP method starts at 60% eluent A. This is not common and I could not find a reason/justification for that in the manuscript. Why not starting at 10% A and employing a steeper gradient? Would not increase the overall run time but better retain most of the metabolites. I do not doubt the results presented (as you have multiple layers to support your putative identifications) but to me the chromatographic method just doesn't look very elegant. Maybe a sentence could be added to highlight future optimization potential?

I agree. We have added a line to the discussion to address this comment (line 347-348).

Line 531: ‚Quality control samples consisting of pooled xenobiotic reactions were analyzed at the beginning, middle, and end of each analytical run.‘ This is not sufficiently detailed (the analytical run could be either 20 injections or 200 e.g.). What was the interval? In HRMS it is generally accepted that about very 5th injection should be a pooled QC. I also miss information how the pooled QC was used to verify robust analytical performance.

We have clarified this (lines 438-441).

Supplemental Table 1 - List of identified metabolites is very comprehensive and useful. I just would like to see information on identification level and a unique identifier (e.g. PubChem ID, ChEBI) added to help secondary use of fellow researcher using cheminformatics. This is crucial as most identifications are not at level 1 (as no reference standards are available) and HRMS non-experts may have issues interpreting the data correctly.

We have added PubChem IDs for as many metabolites as possible (see updated table). We are unable to provide a unique identifier for some metabolites that did not have a PubChem ID (either because the metabolites were not present in PubChem or a specific PubChem ID could not be assigned to that particular entry). We have also clarified this point in the supplemental table.

Some specifics I noted looking at the SI:

Hydroxywarfarin elutes at the same time (26 s) as the native metabolite. Being more polar it

should have more retention on a HILIC column. Any idea why not?

Hydroxynapthalene – not found in neg ESI mode; usually it ionizes better in ESI- than ESI+

The difference in predicted octanol-water partition coefficient (warfarin (2.7 XlogP from Pubchem) and hydroxywarfarin (2.3 XlogP from Pubchem)) is not large enough to shift the retention time on this 5 minute method. There are other compounds where the sulfate/glucuronide caused a shift in RT (Pentachlorophenol 180 seconds on C18 column and the glucuronide form eluting 40 seconds earlier, Acetaminophen 28 seconds on HILIC column and its corresponding OH-sulfate eluting at 138 seconds).

Lines 347-349 were modified to address the comment about expected retention time shifts. We have also modified the entry for hydroxynapthalene in the supplemental table.

Figure S1: It is not clear what ion is depicted. Benzo[a]pyrene is given at m/z 253.1012 while the two hydroxylated metabolites are at m/z 254.0955 and 269.0962? I think there is a mistake; it is also not in line with Table S1. The m/z ratios and retention times do not match.

We have added labels on each extracted ion chromatogram for S1 and also have removed the error and fixed the mass for the sulfate. Thank you for spotting this.

Reviewers' Comments:

Reviewer #3:
Remarks to the Author:
Thank you for giving me the opportunity to look at the second revision of this work.

The authors addressed the remaining issues satisfactory with the exception that they did not add the identification levels in Table S1 as requested. There was no response to this part of the comment allowing me to understand why this was not fixed (original remark: ‚I just would like to see information on identification level and a unique identifier (e.g. PubChem ID, ChEBI) added to help secondary use of fellow researcher using cheminformatics. This is crucial as most identifications are not at level 1 (as no reference standards are available) and HRMS non-experts may have issues interpreting the data correctly.‵.

Reviewer #3 (Remarks to the Author):

Thank you for giving me the opportunity to look at the second revision of this work.

The authors addressed the remaining issues satisfactory with the exception that they did not add the identification levels in Table S1 as requested. There was no response to this part of the comment allowing me to understand why this was not fixed (original remark: ‚I just would like to see information on identification level and a unique identifier (e.g. PubChem ID, ChEBI) added to help secondary use of fellow researcher using cheminformatics. This is crucial as most identifications are not at level 1 (as no reference standards are available) and HRMS non-experts may have issues interpreting the data correctly.‘.

We have added this information to the table and added additional clarification in the methods on identification criteria.
